# Multi-Input data ASsembly for joint Analysis (MIASA): A framework for the joint analysis of disjoint sets of variables

**DOI:** 10.1371/journal.pone.0302425

**Published:** 2024-05-10

**Authors:** Nomenjanahary Alexia Raharinirina, Vikram Sunkara, Max von Kleist, Konstantin Fackeldey, Marcus Weber

**Affiliations:** 1 Department of Mathematics & Computer Science, Freie Universität Berlin, Berlin, Germany; 2 Departement of Modeling and Simulation of Complex Processes, Zuse Institute Berlin, Berlin, Germany; 3 Departement of Visual and Data-Centric Computing, Zuse Institute Berlin, Berlin, Germany; 4 Project Groups, Robert-Koch Institute, Berlin, Germany; 5 Institute of Mathematics, Technical University Berlin, Berlin, Germany; Bocconi University: Universita Bocconi, ITALY

## Abstract

The joint analysis of two datasets X and Y that describe the same phenomena (e.g. the cellular state), but measure disjoint sets of variables (e.g. mRNA vs. protein levels) is currently challenging. Traditional methods typically analyze single interaction patterns such as variance or covariance. However, problem-tailored external knowledge may contain multiple different information about the interaction between the measured variables. We introduce MIASA, a holistic framework for the joint analysis of multiple different variables. It consists of assembling multiple different information such as similarity vs. association, expressed in terms of interaction-scores or distances, for subsequent clustering/classification. In addition, our framework includes a novel qualitative Euclidean embedding method (qEE-Transition) which enables using Euclidean-distance/vector-based clustering/classification methods on datasets that have a non-Euclidean-based interaction structure. As an alternative to conventional optimization-based multidimensional scaling methods which are prone to uncertainties, our qEE-Transition generates a new vector representation for each element of the dataset union X∪Y in a common Euclidean space while strictly preserving the original ordering of the assembled interaction-distances. To demonstrate our work, we applied the framework to three types of simulated datasets: samples from families of distributions, samples from correlated random variables, and time-courses of statistical moments for three different types of stochastic two-gene interaction models. We then compared different clustering methods with vs. without the qEE-Transition. For all examples, we found that the qEE-Transition followed by Ward clustering had superior performance compared to non-agglomerative clustering methods but had a varied performance against ultrametric-based agglomerative methods. We also tested the qEE-Transition followed by supervised and unsupervised machine learning methods and found promising results, however, more work is needed for optimal parametrization of these methods. As a future perspective, our framework points to the importance of more developments and validation of distance-distribution models aiming to capture multiple-complex interactions between different variables.

## Introduction

Real-life processes are usually based on the interaction between distinct variables. For example, the process of gene regulation within cells involves mRNAs and proteins. Nowadays, a large amount of data can be collected for many variables, making their analysis more and more challenging. Current data analysis methods are usually focused on understanding a single interaction pattern. Between multiple variables, multivariate data analysis methods are often used to identify interdependence between the variables. For example, correspondence analysis investigates the patterns related to the statistical independence of two categorical variables where their joint occurrence is given by a contingency table [1, 2]. Measures of interdependence can represent the degree of association between the variables, however, other measures of association may be constructed through external knowledge about the interaction between the variables. For example, the correlation between gene expression and protein expression may represent their degree of association.

The interaction patterns between variables can be represented by a distance matrix containing interaction measures between the sample vector or categories of the variables or between any objects representing them. To analyze data that have Euclidean distance matrix structure, the Schoenberg criterion [3, 4] can be used to recover the results of the low-rank matrix approximation theorem [5] which constructs a new vector representation of the data such that each dimension contains a well-defined pattern of explained variance in each dimension. This process is known as principal component analysis or PCA when directly applied to the data matrix. Correspondence analysis also employs the low-rank matrix approximation by analyzing the patterns of statistical interdependence between variables instead of patterns of variance. For non-Euclidean interaction structure, however, there exist no Euclidean vector representations for the dataset, and thus contemporary methods have been applying non-metric multidimensional scaling methods searching for approximate vector representations that minimize some desired distortion measure between the original distance and the distances in the approximated Euclidean space [6]. This approximation method requires prior selection of the number of dimensions of the approximated Euclidean space and is vulnerable to uncertainties in the optimization process. Therefore, contemporary non-metric multidimensional scaling methods lead to unpredictable alterations (although minimized) of the original information provided by the assembled distance matrix.

Some multivariate data analysis methods have been developed for analyzing multi-block datasets or multi-block distance datasets, which contain data from various sources [7, 8]. These methods, however, do not analyze disjoint variables but rather analyze multiple different feature space representations of one variable. Although the feature space representations are sample measures of other variables, they are only used to explore the patterns of differences within this one variable they represent (e.g., exploring the shared information between different measurements of the same object [9]). For example, one variable can be “cancer cell line”, and each category of cancer cell line can be represented as a row vector including the copy number variations (CNVs) of their genes or their gene expression data. A two-block data can be constructed for the same group of cancer cell lines by stacking the row vector CNVs in one block and gene expression data in another block. Likewise, a two-block distance matrix can be constructed between the same group of cancer cell lines by stacking the pairwise distance between the cancer lines in each block. Most methods for analyzing multi-block data are described as “data fusion” methods. This fusion operates by transforming the non-aligned data blocks into correlation- or variance- or model-based aligned data blocks and then identifies the principal components of these blocks by solving complex extensions of the low-rank matrix approximation problems [8]. All these methods require prior knowledge of the parameters that would work best for the datasets and the number of dimensions (components). Solberg *et al.* proposed the analysis of multi-block distance matrix provided that the distance matrices are Euclidean, by using a multidimensional scaling method extending the low-rank matrix approximation theorem to each distance block and then applying other optimization techniques to find the principal axes vectors that maximize the explained variance across the data-blocks (common components) and within individual blocks (distinct components) [7]. However, the authors found that there is always a trade-off between these common and distinct components, and the term “common” can only refer to either resemblance across blocks (correlation) or explanation of a large part of the observations (variance). The acceptability of all the methods’ trade-offs is then left to the analysts’ better judgment. In both cases, the methods are complex, difficult to interpret, and ignore the possibility of multiple interactions between the categories of variables involved (e.g., by only analyzing the correlation between gene expression within each group of the cancer cell lines, the possible interdependence between gene expression and cancer cell categories is ignored).

Another method widely used in data analysis is clustering (e.g.: [10–12]). This method is focused on finding similarity patterns between samples representing the same variable. For example, two genes may be termed similar if their gene expression levels are similar in magnitudes. Numerous clustering methods, such as the *k*-means [13, 14], the *k*-medoids [15], the agglomerative hierarchical clustering [16] methods, and spectral clustering methods [17], have already been developed. The choice of a suitable clustering method is, however, not universal and largely depends on the specific aim and application of the investigation [10]. For example, the *k*-means algorithm has been found to perform well in the clustering of cancer gene expression data aiming to identify cancer subtypes [18]. In general, however, all clustering methods aim to identify meaningful proximity between the objects in the dataset. This proximity represents the specific patterns that researchers aim to identify and is expressed in terms of distance between the objects. Distance-based clustering methods can be divided into two groups, the Euclidean-distance-based methods and the non-Euclidean distance-based methods. Euclidean-distance-based methods can only perform clustering using a distance that is an Euclidean distance. For example, the Ward clustering method in which the Ward method [19] is used as a linkage method within a hierarchical clustering process, requires the Euclidean distance (although it has also been shown to work for the Manhattan distance [20]). On the other hand, non-Euclidean distance-based methods, such as the *k*-medoids, work for any type of distance. In all cases, Euclidean or non-Euclidean, distance-based clustering methods have also been mostly used to identify single interaction patterns within categories of the same variables belonging to the same feature space.

From the perspective of machine learning (ML), the term “cluster” is substituted with the term “class” but the principle is the same as that of clustering, that is, the aim is to find meaningful classification of the categories of one variable sharing the same feature space representation. Available machine learning methods can be categorized into two major groups: unsupervised, and supervised. To our knowledge, however, they are mostly either Euclidean-distance-based, such as unsupervised Self-Organizing-Maps [21], or Euclidean-vector-based, such as supervised Neural Networks [22–24] and Support Vector Machines [25, 26]. Other methods termed supervised multiple-metric learning attempt to learn complex nonlinear data structures by identifying and merging multiple local metrics that fit the best classification structure in the training data [27]. Despite that these methods seem based on multiple interaction measures, broadly termed as multiple metrics, they also primarily require Euclidean-vector representations of the items to be classified. Therefore, most currently available machine learning methods are also not directly accessible for the joint analysis of disjoint sets of variables.

In this paper, we propose MIASA, a holistic framework that involves two major contributions. The first contribution is that the MIASA framework can be tailored to multiple distinct variables describing the same phenomenon or process and investigate multiple interaction patterns between them. The variables can be of any type (categorical, continuous, or other) as long as the interaction measures, such as similarity vs. association, can be properly defined between them and assembled into one distance matrix (which is likely a non-Euclidean one). The second contribution is that our framework includes a novel Euclidean embedding method (qEE-Transition) that strictly preserves the ordering of distances. It is designed to combine the similarity and association distances information into a single Euclidean distance and subsequently applies an Euclidean-distance-based clustering method or vector-based machine learning methods to simultaneously observe the similarity and associations between the objects of interest. We suggest our qEE-Transition method for dealing with the assembled non-Euclidean distance matrix as an alternative to the conventional non-metric multidimensional method which does not provide well-defined patterns of explained variance and is unlikely to preserve the information carried by the assembled distance matrix. Since clustering or machine learning algorithms are also often based on optimization criteria, we preferred not to add another uncertainty in the clustering/classification process.

Our manuscript is organized as follows. The next section, Materials and methods, is dedicated to the main components of the MIASA framework and problems designed for simulation experiments, the second section includes the results of our simulation experiments (Results), and we finalize our paper with the sections Discussion and Conclusion. Our Materials and methods section is subdivided into several subsections. The first subsection is a formal formulation of the problem intended to be solved for two disjoint sets of variables. The second subsection describes the construction of the assembled distance matrix that represents the joint interactions between the variables. The third and fourth subsections cover the description of the qEE-Transition that enables the construction of a joint Euclidean space for the disjoint variables. The fourth subsection describes the clustering, machine learning, and data-visualization methods integrated into the MIASA framework. The fifth subsection formalizes three potential problems, and the remaining subsections describe the evaluation of clustering results and the implementation of our framework. Next, our Results includes the results of our simulation experiments. First, we present a snapshot of the identification of cluster memberships for the three problem showcases described in Materials and methods, then we present the result of a thorough evaluation of the clustering results with qEE-Transition vs. without, and finish this section with an experiment on machine learning methods. Finally, sections Discussion and Conclusion summarize our work and present future potential research directions.

## Materials and methods

### Problem formulation

In the MIASA framework, we consider two independent datasets X and Y containing m,n∈N\{0,1} samples or timeseries representing specific objects. We combine the two datasets in one dataset D, denoted

$$\begin{array}{c} D=X\cup Y,\;\text{such}\;\text{that}\;X=\{x_{1},\ldots ,x_{m}\}\;\text{and}\;Y=\{y_{1},\ldots ,y_{n}\}. \end{array}$$ (1)

where **x**_•_ and **y**_•_ are vectors, matrices, or other data representations, containing several observations of a random variable or time-dependent observation of a specific variable.

Let $d_{X}$ and $d_{Y}$ be the similarity distance between the elements of X and Y, respectively, such that at least one them is an Euclidean distance. Furthermore, let $d_{XY}$ be the association distance between elements of the different sets, which is an arbitrary positive function that is not zero everywhere.

**Fig 1:**
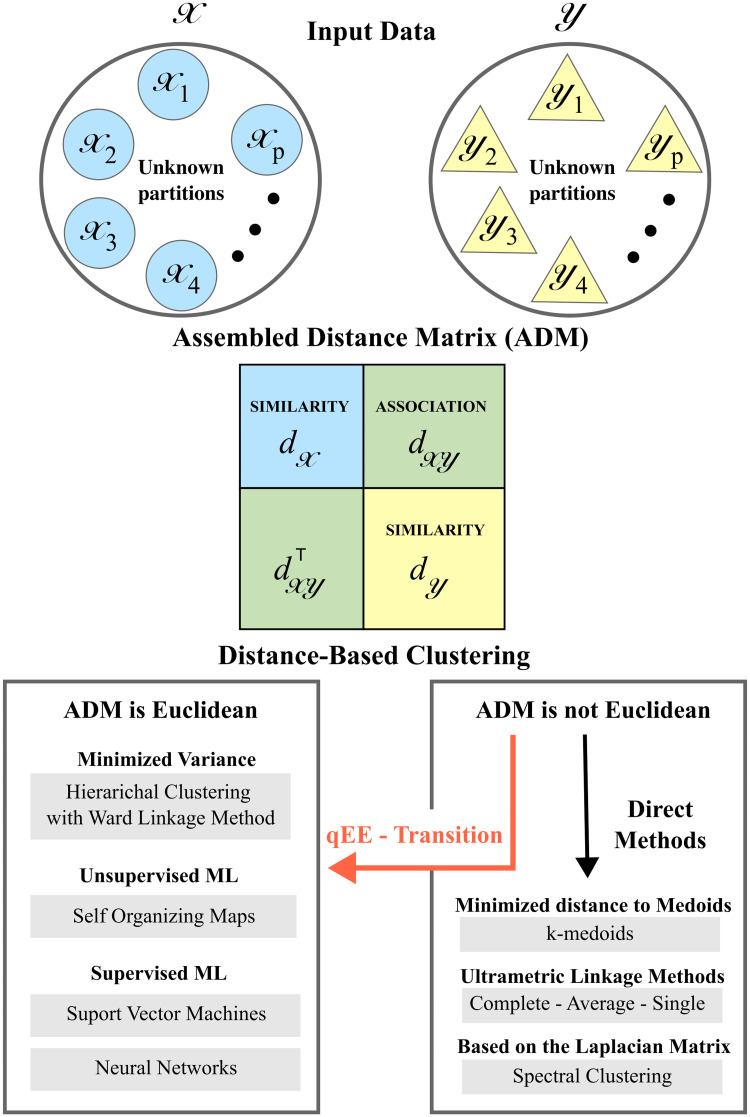
Concept of the MIASA framework. Starting from two separated datasets X and Y, then assembling similarity distances $d_{X}$ and $d_{Y}$, and association distances $d_{XY}$. Finally, clustering with or without the qEE-Transition.

The MIASA framework aims to identify the patterns of similarity and association between the elements of D through the cluster memberships corresponding to the distance triplets $\left(d_{X},d_{Y},d_{XY}\right)$. The framework is conceptualized in Fig 1 and the specific components are described in the sections that follow.

**NB:** We note that X and Y might contain representations of more than one variables. What is important is the similarity distance and the association distance have to be properly defined.

### Assembled similarity and association distance

Let X and Y be two separated datasets and D be their union as described in Problem formulation. The choice of similarity distance $d_{X}$ and $d_{Y}$ between the elements of the same dataset should correspond to the target clustering membership. It thus depends on the information that we intend to extract from the dataset which needs to be properly conceptualized.

Before going to the next step, at least one of the similarity distances $d_{X}$ and $d_{Y}$ must be Euclidean; however, an extra step may be performed when the problem requires a non-Euclidean similarity distance is necessary (see qEE-Transition: Dealing with a Non-Euclidean similarity). An Euclidean similarity distance may be defined as the Euclidean distance between feature transformations of the elements of X and/or Y. That is, we denote

$$\begin{array}{c} U\left(X\right)=\{u\left(x_{1}\right),\ldots ,u\left(x_{m}\right)\}\;\text{and/or}\;V\left(Y\right)=\{v\left(y_{1}\right),\ldots ,v\left(y_{n}\right)\}, \end{array}$$ (2)

where *u* and *v* are feature transformations corresponding to the research concept. The pairwise similarity distance between the elements of our dataset is thus given as follows, for every *q*, *q*′ ∈ {1, …, *m*} and *l*, *l*′ ∈ {1, …, *n*}

$$\begin{array}{c} d_{X}\left(x_{q},x_{q^{\prime }}\right)=\left|\left|u\left(x_{q}\right)-u\left(x_{q^{\prime }}\right)\right|\right|\;\text{and/or}\;d_{Y}\left(y_{l},y_{l^{\prime }}\right)=\left|\left|v\left(y_{l}\right)-v\left(y_{l^{\prime }}\right)\right|\right|, \end{array}$$ (3)

where ||•|| represents the Euclidean norm.

The concept of association may be used interchangeably with the concept of similarity. Here, however, the term association distance is solely defined as the distance between elements of the different datasets X and Y (Problem formulation). Let $d_{XY}$ be the association distance associated with each pairs $(x_{q},y_{l})\in X\times Y$. Our framework requires that $d_{XY}$ is positive and non-identically zero everywhere. Here again, the association distance depends on the definition of cluster membership that we are aiming to re-construct. That is, it may be any problem-tailored measure of interaction between variables.

Following the above definitions, the similarity distance and association distance are not required to carry the same information. Therefore, the MIASA framework combines the disjoint datasets by incorporating different pieces of information describing the phenomenon represented by the variables.

### qEE-Transition: An Euclidean distance that joins disjoint datasets

The key component of MIASA is that the clustering process is always performed using an Euclidean distance that joins the similarity and association distance information. This Euclidean distance, denoted $\Lambda_{X\cup Y}$, is derived from a qualitative Euclidean embedding (qEE) of all the elements of D=X∪Y under the condition that at least one of the similarity distances is Euclidean (see [28]). The qEE is based on the existence of *c*_1_, *c*_2_ > 0 and *c*_3_ > 0 (dependent on *c*_1_ and *c*_2_) such that for every *q*, *q*′ ∈ {1, …, *m*} and *l*, *l*′ ∈ {1, …, *n*}

$$\begin{array}{cc} \Lambda_{X\cup Y}\left(x_{q},x_{q}\right) & =0,\;\Lambda_{X\cup Y}\left(y_{l},y_{l}\right)=0, \end{array}$$ (4)

$$\begin{array}{cc} \Lambda_{X\cup Y}\left(x_{q},y_{l}\right) & =\Lambda_{X\cup Y}\left(y_{l},x_{q}\right), \end{array}$$ (5)

$$\begin{array}{cc} \Lambda_{X\cup Y}\left(x_{q},x_{q^{\prime }}\right) & =\sqrt{d_{X}{\left(x_{q},x_{q^{\prime }}\right)}^{2}+c_{3}\zeta },\;q\neq q^{\prime }, \end{array}$$ (6)

$$\begin{array}{cc} \Lambda_{X\cup Y}\left(y_{l},y_{l^{\prime }}\right) & =\sqrt{d_{Y}{\left(y_{l},y_{l^{\prime }}\right)}^{2}+c_{3}\zeta },\;l\neq l^{\prime }, \end{array}$$ (7)

$$\begin{array}{cc} \Lambda_{X\cup Y}\left(x_{q},y_{l}\right) & =\sqrt{d_{XY}{\left(x_{q},y_{l}\right)}^{2}+c_{3}\zeta }, \end{array}$$ (8)

where

$$\zeta =\{\begin{array}{ll} \zeta_{x_{1}}=\underset{\begin{array}{c} x\in X\\ y\in Y \end{array}}{\text{max}}\sum_{\begin{array}{c} y\in Y\\ x\in X \end{array}}\left|\frac{d_{X}{\left(x,x_{1}\right)}^{2}+d_{XY}{\left(y,x_{1}\right)}^{2}-d_{XY}{\left(x,y\right)}^{2}}{2}\right|, & \text{if}\;\text{only}\;d_{X}\;\text{is}\;\text{Euclidean},\\ \zeta_{x_{1}}, & \text{if}\;d_{X}\;\text{and}\;d_{Y}\;\text{are}\;\text{Euclidean},\\ \zeta_{y_{1}}=\underset{\begin{array}{c} x\in X\\ y\in Y \end{array}}{\text{max}}\sum_{\begin{array}{c} y\in Y\\ x\in X \end{array}}\left|\frac{d_{Y}{\left(y,y_{1}\right)}^{2}+d_{XY}{\left(x,y_{1}\right)}^{2}-d_{XY}{\left(x,y\right)}^{2}}{2}\right|, & \text{if}\;\text{only}\;d_{Y}\;\text{is}\;\text{Euclidean}, \end{array}$$ (9)

Additionally, a theoretical point of origin **o** can be added to encode the information on the magnitudes of the elements of D. That is, the qEE can be calibrated to carry the following information

$$\begin{array}{c} \Lambda_{X\cup Y}{\left(x_{q},o\right)}^{2}={\left|\left|x_{q}\right|\right|}^{2}+c_{3}\zeta \;\text{and}\;\Lambda_{X\cup Y}{\left(y_{l},o\right)}^{2}={\left|\left|y_{l}\right|\right|}^{2}+c_{3}\zeta . \end{array}$$ (10)

The use of the theoretical point **o** (Eq (10)) is optional and can be removed entirely for both datasets X and Y or partially for only one of the dataset. It is particularly useful when the magnitude of the elements of X or Y carries information that we want to preserve in the Euclidean embedding.

Any triplet (*c*_1_, *c*_2_, *c*_3_) satisfying Eq (11) bellow is a solution of Eqs 4 to 8

$$\begin{array}{c} \left\{ \begin{array}{c} c_{1}\geq K,\\ c_{2}\geq K^{\prime },\\ 2+c_{1}+c_{2}-c_{3}=0, \end{array}\right. \end{array}$$ (11)

where *K* and *K*′ are some non-negative numbers that depend on *c*_1_, *c*_2_, and on the range values of the similarity and association distances. This result is derived in [28], under the conditions that at $d_{X}$ or $d_{Y}$ is Euclidean, using the Geršgorin circle theorem [29] and the Schoenberg criterion [3, 4]. This criterion is a necessary and sufficient condition for $\Lambda_{X\cup Y}$ to be an Euclidean distance. Accordingly, **Algorithm** 1 converges to the triplet (*c*_1_, *c*_2_, *c*_3_) satisfying Eq (11). [!h]

**Algorithm 1:** An algorithm that finds a solution for Eq (11)

**1** *c*_1_ ← 1/2;

**2** *c*_2_ ← 2;

**3** $c_{3}\leftarrow \frac{\left(2c_{1}+c_{2}\right)-\frac{2c_{2}}{m+n}}{1-\frac{1}{m+n}}$ ;

**4** compute $\Lambda_{X\cup Y}$;

**5** **while** *Schoneberg criterion on* $\Lambda_{X\cup Y}$ *FALSE* **do**

**6**  *c*_1_ ← *c*_2_;

**7**  *c*_2_ ← 2*c*_1_;

**8**  *c*_3_ ← 2 + *c*_1_ + *c*_2_;

**9 end**

We adopted the initialization of algorithm 1 because they were obtained from previous trials and errors and proved to solve previous problems without necessarily being a solution of Eq (11) [28]. Nevertheless, *c*_1_, *c*_2_, *c*_3_ can also be initialized with random positive numbers. In most of the simulations presented here, the provided initialization solves the qEE problem, however, for random initialization between 0 and 1, we found that the algorithm finds a solution within 4 iterations. The algorithm 1 enables us to find a feasibility region for the existence of the Euclidean embedding although it is not optimal.

### qEE-Transition: Dealing with a non-Euclidean similarity

As mentioned above, our framework combines two similarity distances, $d_{X}$ and $d_{Y}$, and one association distance $d_{XY}$ into one Euclidean distance $\Lambda_{X\cup Y}$. The construction of $\Lambda_{X\cup Y}$ requires that at least one of the similarity distances is an Euclidean distance. However, some problems might require using non-Euclidean similarity distance for both datasets X and Y because they might provide a more meaningful conception of cluster membership. In such a case, we can use the qEE method to transform one of the similarity distances into an equivalent Euclidean distance as described in the previous section, and then use the derived Euclidean distance as $d_{X}$. This means that for every *q*, *q*′ ∈ {1, …, *m*} and *l*, *l*′ ∈ {1, …, *n*}, we redefine the similarity distances as follows

$$\begin{array}{c} d_{X}\left(x_{q},x_{q^{\prime }}\right)=\Lambda_{X\cup \tilde{X}}\left({\tilde{x}}_{q},{\tilde{x}}_{q^{\prime }}\right)\;\text{and/or}\;d_{Y}\left(y_{l},y_{l^{\prime }}\right)=\Lambda_{Y\cup \tilde{Y}}\left({\tilde{y}}_{l},{\tilde{y}}_{l^{\prime }}\right), \end{array}$$

where $\tilde{X}=X$ is the dataset duplicate with elements ${\tilde{x}}_{q}=x_{q}$ relabeled to make the distinction between the original dataset and the duplicate, and similarly for $\tilde{Y}$. The metrics $\Lambda_{X\cup \tilde{X}}$ and $\Lambda_{Y\cup \tilde{Y}}$ are Euclidean distances obtained from the qEE of $X\cup \tilde{X}$ and $Y\cup \tilde{Y}$, respectively, and are constructed as follows.

Let $d_{X}^{\left(M_{1}\right)}$ and $d_{Y}^{\left(M_{2}\right)}$ be the two non-Euclidean distance that is representative of the similarity distance between the elements of the same dataset. Furthermore, let $d_{X}^{\left(Eucl\right)}$ and $d_{Y}^{\left(Eucl\right)}$ be any arbitrary Euclidean distance between the objects, which are only used as a placeholder and does not have to carry any meaningful information. Then, the Euclidean distances $\Lambda_{X\cup \tilde{X}}$ and $\Lambda_{Y\cup \tilde{Y}}$ are constructed using the same procedure as for obtaining $\Lambda_{X\cup Y}$ in the previous section. That is, $\Lambda_{X\cup \tilde{X}}$ is obtained by constructing the Euclidean metric space $\left(X\cup \tilde{X},\Lambda_{X\cup \tilde{X}}\right)$ using the distance triplets

$$\begin{array}{c} (d_{X}=d_{X}^{\left(Eucl\right)},d_{\tilde{X}}=d_{X}^{\left(M_{1}\right)},d_{X\tilde{X}}=d_{X}^{\left(M_{1}\right)}), \end{array}$$

and similarly, $\Lambda_{Y\cup \tilde{Y}}$ is obtained using the distance triplets

$$\begin{array}{c} (d_{Y}=d_{Y}^{\left(Eucl\right)},d_{\tilde{Y}}=d_{Y}^{\left(M_{2}\right)},d_{Y\tilde{Y}}=d_{Y}^{\left(M_{2}\right)}), \end{array}$$

In the above triplets distances, $d_{X\tilde{X}}$ and $d_{Y\tilde{Y}}$ can also be replaced by arbitrary non-negative function. Then, from the Euclidean embedding, we extract the following information

$$\begin{array}{c} \Lambda_{X\cup \tilde{X}}\left({\tilde{x}}_{q},{\tilde{x}}_{q^{\prime }}\right)=\sqrt{d_{X}^{\left(M_{1}\right)}{\left(x_{q},x_{q^{\prime }}\right)}^{2}+c_{3}\left(X\cup \tilde{X}\right)\;\zeta \left(X\cup \tilde{X}\right)}, \end{array}$$

and/or

$$\begin{array}{c} \Lambda_{Y\cup \tilde{Y}}\left({\tilde{y}}_{q},{\tilde{y}}_{q^{\prime }}\right)=\sqrt{d_{Y}^{\left(M_{2}\right)}{\left(y_{q},y_{q^{\prime }}\right)}^{2}+c_{3}\left(Y\cup \tilde{Y}\right)\;\zeta \left(Y\cup \tilde{Y}\right)}, \end{array}$$

where *c*_3_(•)*ζ*(•) are obtained using the procedure described in the previous section but depend on the specific datasets. In this way, $\Lambda_{X\cup \tilde{X}}$ and $\Lambda_{Y\cup \tilde{Y}}$, specifically restricted on the elements of $\tilde{X}$ and $\tilde{Y}$, carry the same information as the non-Euclidean distances $d_{X}^{\left(M_{1}\right)}$ and $d_{Y}^{\left(M_{2}\right)}$ with which we aim to use for clustering.

### Clustering/classification and lower dimensional visualization

Finally, the MIASA framework is completed by clustering the elements of the dataset D using their Euclidean embedding in the metric space $\left(D,\Lambda_{X\cup Y}\right)$ which is obtained from the qEE-Transition. Because $\Lambda_{X\cup Y}$ is the square root of a positive scaling of the squared similarity and association distances (Eqs 4 to 8)), the true clusters of the metric space $\left(D,\Lambda_{X\cup Y}\right)$ should be equal to the true clusters of the non-Euclidean space $\left(D,d_{X}/d_{Y}/d_{XY}\right)$. An Euclidean-distance-based clustering method can be used to identify the clustering patterns associated with $\Lambda_{X\cup Y}$ but only a non-Euclidean-distance-based clustering method can be used to identify clustering patterns associated with $d_{X}/d_{Y}/d_{XY}$.

As listed in the introduction, Euclidean-distance-based clustering methods include the Ward clustering method minimizing the total within-cluster variance [19] and the *k*-mean clustering method minimizing the total square distance to cluster center points or centroids [13, 14]. Both of these methods are well known in the clustering research community and each of them is based on a reasonable criterion for cluster membership, thus, choosing between them is not evident. Here we need to account for a technical aspect of the qEE-Transition, which by construction, tends to embed the dataset in high dimensional space such that the Gramian matrix of the embedded points is almost full rank. However, it has been shown that the *k*-mean method generally performs poorly on high-dimensional data (see [30] for a review). After performing several tests, we confirmed that the *k*-mean method (currently implemented in Python) was incompatible with the qEE-Transition. Conversely, the Ward clustering method seemed to work well in combination with qEE-Transition, thus we advise using the Ward clustering method instead of the *k*-means method.

We also integrated several machine learning approaches designed for supervised and unsupervised data classification to complement the qEE-Transition within MIASA. As an unsupervised ML method, we integrated the Self-Organizing-Maps [21] which is an Euclidean-distance-based learning algorithm consisting of initializing a group of vectors (nodes) that has the same number of our desired cluster numbers and iteratively finding the vector that is the closest to each sample vectors of the datasets (the best matching unit or BMU) and moving these BMUs closer and closer to their most similar sample vectors (following some learning rate parameter) until the number of maximum iteration is achieved. The result of the Self-Organizing-Maps, thus, depends on the initialization, the maximal number of iterations, and the learning rate. In the Python implementation of the Self-Organizing-Maps [31], the two latter parameters are left to the better judgment of the users. As supervised machine learning methods, we integrated a Neural Network [22–24] and a Support Vector Machines method [25, 26] which are both Euclidean-vector-based. Supervised machine learning requires prior knowledge of a certain proportion of the true cluster membership, this data is termed training dataset. The Neural Network training process assumes that the training input vectors (training cluster members) can be transformed into the output training (training cluster labels) through a composition of one or more functions (feedforward multi-layer perceptron). Each layer is represented as a collection of nodes where the nodes of the first layer are the input vectors and the nodes of the next layer contain the output of the first function operations which then become inputs for the next function operation, and so on until reaching the last layer which contains the output training. Each node of a given layer is assigned a weight representing its contribution to the function that leads to the next layer, and each weight is considered to various degrees depending on a chosen activation function. The iteration steps of the Neural Network then aim to find the optimal weight configurations of this multi-layer perceptron by minimizing the distortion between the calculated least square error loss between the calculated output and the training output or by minimizing a cross-entropy estimate. After the final estimation of the weights of the multi-layer perceptron, the remaining data is used to test the performance of the Neural Network. The output of the Neural Network depends on parameters such as learning rates used to update the weights along the gradient of the loss function, the number of functions used (hidden layers), the number of iterations of the algorithm, and the number of training vectors. The Support Vector Machines method is also supervised, however, instead of using the notion of functions, it achieves its learning process by searching for the hyperplane that maximizes the separation (margin) between the points belonging to different classes [32]. It achieves a fast computing performance by using the “kernel trick” [33] in which direct computation of the dot product in the algorithm is replaced by a linear function of the kernel thereby reducing computational complexity [33]. The performance of this method depends on the number of training vectors, and the maximal number of iterations, and additionally requires an educated guess on the kernel function.

In all implementations of the subsequent clustering/classification following qEE-Transition, the theoretical point of origin **o** (Eq (10)) is used in our examples but is removed from the clustering process because in several experiments it was assigned to one cluster separated from all the other points.

For evaluating the contribution of the qEE-Transition combined with the Ward clustering, we selected five non-Euclidean-distance-based clustering methods: agglomerative hierarchical clustering with a complete, average, and single linkage which are well-known ultrametric linkage methods [34], the *k*-medoids method which is analogous to the *k*-means by replacing the notion of centroid with the notion of medoids (representatives object of the dataset achieving a minimal total distance to cluster members), and the spectral clustering procedure based on Laplacian-Matrix well know in graph theory [17].

Concerning data visualization, our qEE-Transition is currently incompatible with linear visualization methods such as orthogonal projections due to the high dimensionality of the Euclidean embedding as mentioned above. Thus, the visualization of the results is useful for summarizing the findings but is currently not the primary focus of MIASA. Orthogonal projection might not be appropriate because *c*_3_*ζ* (Eqs 4–10) introduces artificial variance in the dataset. The principal orthogonal axes are thus unlikely to be sufficient for a qualitative observation of the original data variance since the pairwise distances are distributed across all the dimensions of the embedding space. Instead of focusing on visualizing the pairwise relationship between the elements of the datasets, we focused on the visualization of cluster membership. Therefore, for our simulated datasets here, we used UMAP [35] or t-SNE [36] projections, which are non-linear dimension reduction method that performs well for the visualization of high-dimensional datasets.

### Potential applications

To demonstrate our approach, we considered three types of simulated datasets and their associated true cluster memberships and chosen similarity and association distances.

#### Problem 1: Clustering data into distinct families of distributions

For this dataset, we considered four families of distributions: the normal, the uniform, the Pareto, and the Poisson distribution. Then, for each family of distributions, we considered five different distributions based on different parameters. From each distribution, we generated 25 sample vectors that we included in the dataset X and a duplicate of 25 sample vectors that we included in the dataset Y (each sample vector contained 300 observations). The union of the datasets, D, thus included 4 × 25 × 2 sample vectors. We then assumed that the true cluster membership corresponds to the family of distributions and aimed to predict four clusters.

The histograms of the sample vectors differentiate them from one another and the shapes of the distributions are often similar for distributions belonging to the same family. Since MIASA attempted to recover the family of distributions, we assumed that the two similarity distances $d_{X}$ and $d_{Y}$, are the Euclidean distances between the heights of numerical histograms of the sample vectors (computed with 10 bins for all histograms). Following our notations in Assembled Similarity and Association distance, the histogram transformation is the feature transformation of the sample vector in this case.

The association distance can be measured by any statistic that reflects the differences in the distributions. For example, an association distance can be defined using the p-value of the Kolmogorov-Smirnov (KS) test denoted *p*_*KS*_. The test corresponds to the null hypothesis that two samples come from the same distribution. As a result, the smaller *p*_*KS*_, the stronger we reject the null hypothesis. Therefore, to predict two samples of the same distribution, we computed the following association distance

$$\begin{array}{c} d_{XY}\left(x_{q},y_{l}\right)=\epsilon +1-p_{KS}\left(x_{q},y_{l}\right), \end{array}$$ (12)

where *ϵ* is a small positive number ensuring that $d_{XY}$ is non zero.

#### Problem 2: Clustering data into distinct correlated random variables

For this dataset, we assumed that each true cluster contains sample vectors drawn from a bivariate normal distribution. Furthermore, we considered 10 different bivariate normal distributions. We generated 25 sample vectors, each of which was composed of 300 observations. The first dimension of each sample vector is then assigned to the dataset X and the second dimension is assigned to Y. The dataset D thus contained 10 × 25 × 2 elements and our framework aimed to predict 10 clusters corresponding to the original distributions.

For the similarity distance, we compared the marginal distributions of the correlated sample vectors. We also used an Euclidean similarity distance for the two datasets X and Y. Accordingly, the pairwise similarity between the elements of each set X and Y is given by the Euclidean distance between the empirical cumulative distribution function of the sample vectors. This information can always be extracted without prior knowledge about the correlation between the elements of X and Y. The empirical cumulative distribution function is thus the feature transformation corresponding to the similarity distance used in this example dataset.

For the association distance, the problem at hand is to identify the correlation between the sample vectors in different datasets. Therefore, any measure of correlation can be used. Here, we used the absolute value of the Spearman rank correlation coefficient (*ρ*(**x**_*q*_, **y**_*l*_)) to identify positive or negative non-linear correlations between (**x**_*q*_ and **y**_*l*_) as follows

$$\begin{array}{c} d_{XY}\left(x_{q},y_{l}\right)=\epsilon +1-\left|\rho \left(x_{q},y_{l}\right)\right|, \end{array}$$ (13)

where *ϵ* is a small positive number ensuring that $d_{XY}$ is non zero everywhere.

#### Problem 3: Clustering mRNA time-course data into regulatory model classes

For this dataset, we represented each object by the time-course of the three moments: the mean, the variance, and the skewness (concatenated or stacked on a matrix) generated by a particular two-gene regulatory network simulating the time courses of mRNA counts for each gene (denoted A and B). We considered three types of two-gene interaction models [37]: the no-interaction model (“No-I”) in which the two genes are regulated independently of each other, the mono-directional interaction model (“Mono-I”) in which gene B actively down-regulates the gene A and bidirectional interaction model (“Bi-I”) in which the two genes regulate each other. For each model, we generated 25 time-course of the moments, and each time-course was empirically calculated from 4000 stochastic time-courses of mRNA counts for each gene. All mRNA A from each model were then assigned to the dataset X and all mRNA B were assigned to the dataset Y and thus the union of the datasets contained 3 × 25 × 2 time-course of moments. To identify regulation patterns, we assumed that interacting genes (Mono-I and Bi-I models) corresponded to the same true cluster and the non-interacting genes (No-I model) corresponded to different true clusters. The dataset is thus composed of 4 different true clusters and our framework was calibrated to predict them.

To make the time courses comparable, we applied min-max normalization as a feature transformation. Then, we considered that all similarity distances are Euclidean and computed as the Euclidean distance between the normalized time courses.

Finding a suitable statistic representing regulation between genes is still an ongoing dilemma [38] and one of the main problems is that it is difficult to separate “correlation” in gene levels from causal mechanisms such as “regulation” between the involved genes [37]. Here, we used the Granger causality concept [39] as it was used in previous gene regulation inference as a proxy for the direction of regulation [40]. Since we needed a statistic, we used the *p*-value of the Granger causality (*p*_*G*_) test, having the null hypothesis that the first vector does not Granger cause the second one or vice-versa. We thus used the following association distance

$$\begin{array}{c} d_{XY}\left(x_{q},x_{l}\right)=\epsilon +\text{mean}\{p_{G}(\Delta x_{q}{|}_{w},\Delta y_{l}{|}_{w}):w=\text{mean},\text{variance},\text{skewness}\}, \end{array}$$ (14)

where *ϵ* is a small positive number ensuring that $d_{XY}$ is non-zero, and the sign |_*w*_ indicates that the time course vector is restricted to *w*, Δ is a differencing transformation of the time-courses ensuring that they are stationary as required by the test [41, 42].

### Performance evaluation

To evaluate the performance of the MIASA framework, we compared the clustering results obtained with the qEE-Transition vs. without it. One of the most common indexes used for clustering validation when the ground truth is known is the adjusted rand index or ARI [43–46]. The ARI score is a total measure of the agreement between the true and the predicted cluster membership of each pair of objects relative to a completely random pairing and it is computed as follows

ARI(T,P)=Index-mean(Index)max(Index)-mean(Index)=∑i,j(Nij2)-∑i,j(Ni•2)(N•j2)/(N2)12[∑i(Ni•2)+∑j(N•j2)]-∑i,j(Ni•2)(N•j2)/(N2),

where $T=\{T_{i},\;i=1,\ldots ,I\}$ is the true cluster partition and $P=\{P_{j},\;j=1,\ldots ,J\}$ is the predicted cluster partition, *N*_*ij*_ is the number of pairs objects that simultaneously belong to cluster $T_{i}$ and $P_{j}$, *N*_*i*•_ = ∑_*j*_*N*_*ij*_, *N*_•*j*_ = ∑_*i*_*N*_*ij*_, and *N* = ∑_*i*,*j*_*N*_*ij*_.

For evaluating cluster memberships in the MIASA framework, we take into account that the partitioning separating the dataset X from the dataset Y is known since they are disjoint datasets to begin with. Additionally, by the construction of our disjoint datasets, there might be some known pairing patterns between the elements of X and Y (this is the case of our three examples in Potential applications). For example, the dataset X is partitioned as $\left\{X_{1},\ldots ,X_{p}\right\}$ and the dataset Y is partitioned as $\left\{Y_{1},\ldots ,Y_{p}\right\}$ such that each pair $\left(X_{k},Y_{k}\right)$ corresponds to the same data collection procedure. Therefore, we used the following accuracy measure

$$\begin{array}{c} \bar{\text{ARI}}=\text{mean}\{\text{ARI}\left(T_{X},P_{X}\right),\;\text{ARI}\left(T_{Y},P_{Y}\right),\;\text{ARI}\left(T_{X_{•}\times Y_{•}},P_{X_{•}\times Y_{•}}\right)\}, \end{array}$$ (15)

where $T_{X}$ is the true partition for X, $P_{X}$ is the predicted partition for X, $T_{Y}$ is the true partition for Y, $P_{Y}$ the predicted partition for Y, $T_{X_{•}\times Y_{•}}$ is the true partition pairing, and $P_{X_{•}\times Y_{•}}$ is the predicted partition pairing.

### Implementation and simulations

The MIASA framework was implemented in Python mainly using SciPy [47], NumPy [48], and scikit-learn [49]. All codes are available at https://github.com/AlexiaNomena/MIASA. The main results of this manuscript were computed from Jupyter Notebooks (https://jupyter.org) and a snakemake workflow (https://snakemake.readthedocs.io/en/stable/) pipeline is provided to enable the users to apply the framework on their datasets. The simulations required for performance evaluation were performed on the high-performance computing (HPC) cluster at ZEDAT, Freie Universität Berlin [50]. Figures were finalized with Inkscape 1.2.

## Results

In this section, we present the results of our simulation experiments. The simulations are designed to assess the performance of MIASA as compared with the analog non-metric clustering framework. We first simulated an example of clustering obtained from a random sample dataset of each of the three dataset types (Potential applications).

### Identified clusters versus true clusters

To illustrate the type of results that can be obtained using MIASA, we performed one test simulation for each dataset type. Each predicted cluster is displayed in separate panels to facilitate the comparison between predicted and true clusters. We considered the two cases of similarity distance using the same datasets in both of them. That is, in the first case we considered that both $d_{X}$ and $d_{Y}$ are Euclidean (as described in Potential applications), and in the second one, we considered that both of them are *L*^3^-norms between the features to show how to use the method for non-Euclidean similarity distance (although there is no conceptual motivation for this).

**Fig 2:**
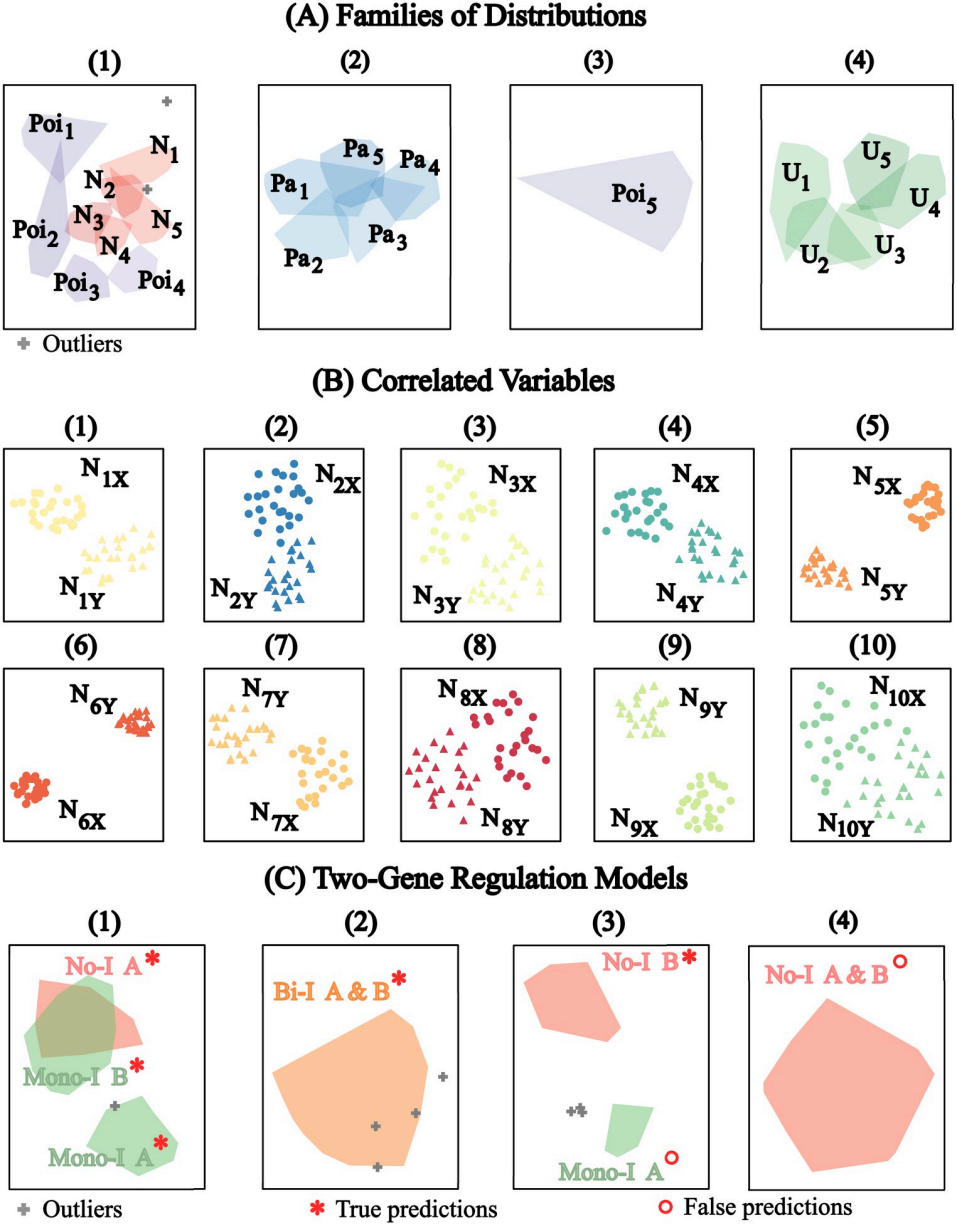
MIASA-prediction for Euclidean similarity distance. A: UMAP projections of predicted families of distributions (in separate panels 1–4) versus convex hulls of the data points belonging to the different distributions: Poisson (Poi_1_ to Poi_5_), Normal (N_1_ to N_5_), Pareto (Pa_1_ to Pa_5_), and Uniform (U_1_ to U_5_). B: UMAP projections of predicted clusters (in separate panels 1–10) versus samples of bivariate normal distributions, first dimensions (N_1X_ to N_10X_) and second dimensions (N_1Y_ to N_10Y_). C: UMAP projections of predicted clusters (in separate panels 1–4) versus true gene regulation patterns between gene A and gene B (convex hulls of data point representations): No-I A, No-I B, Mono-I A & B, and Bi-I A & B. True and False predictions are only evaluated for the pairs of genes belonging to the same models.

The results for the Euclidean similarity distance are shown in Fig 2 using UMAP visualization. For Data Type 1, the predicted clusters showed a good agreement with the true cluster representation of the family of distributions. The results are shown in Fig 2A and each predicted cluster is shown in different panels from (1) to (4). The five individual distributions simulated for each family of distribution are also shown as the convex hull of the projections of their sample vectors. The family of Uniform and Pareto Distributions were identified with 100% accuracy in panels (2) and (4), respectively. The family of Normal and Poisson distributions were combined into a single cluster in panel (1) except for the 5^th^ Poisson distribution which was assigned to cluster number (3). However, in panel (1), the Normal distributions are grouped while the Poison distributions surround them. Additionally, Fig 2A shows that the individual distributions can still be well distinguished on the UMAP projections. For Data Type 2, representing samples of correlated random variables, we also found that the sample correlated bivariate variables were identified with 100% accuracy (Fig 2B). Interestingly, the UMAP projections of each cluster indicated a clear separation between the sample vectors representing the first dimension (labeled X) and the correlated sample vectors’ second dimension (labeled Y). Notably, the 6^*th*^ cluster showed the strongest separation between the sample vectors, and the 10^*th*^ cluster showed the weakest separation. We examined the average magnitude of Spearman’s rank correlation constant for each of the distributions for each predicted cluster and found that the highest was 35% higher than the mean, while the lowest was 7% higher than the mean. These bounds effectively belonged to the 6^*th*^ and 10^*th*^ clusters, respectively. However, the average deviation from the mean of Spearman’s rank coefficient did not always follow the separation pattern, for example, it was 29% for cluster 8 but only 12% for cluster 9 although they show the opposite separation pattern (Fig 2). Thus, the UMAP patterns need to be interpreted cautiously. Lastly, for Data Type 3, representing three models of two-gene interactions, we already have the prior knowledge that the genes in the separate models could never interact with each other because they can also be considered to come from completely different experiments. Therefore, the prediction combining pairs of genes that do not belong to the same model can be ignored as this pattern is only a coincidence. Fig 2C shows the patterns of regulation between the genes it predicted all the interaction models but with a few false predictions. Firstly, the no-interaction model (No-I) was partially identified with gene A predicted in cluster 1 and gene B predicted in cluster 3 but both gene A and gene B predicted in cluster 4. The mono-directional model (Mono-I) was predicted in the 1^*th*^ cluster with a clear separation between gene A and gene B, but also with a false positive position of gene B in cluster 3 and the outliers in cluster 2. The bidirectional interaction model (Bi-I) was well predicted in cluster 2 (with a few of them as outliers in clusters 1 and 3).

**Fig 3:**
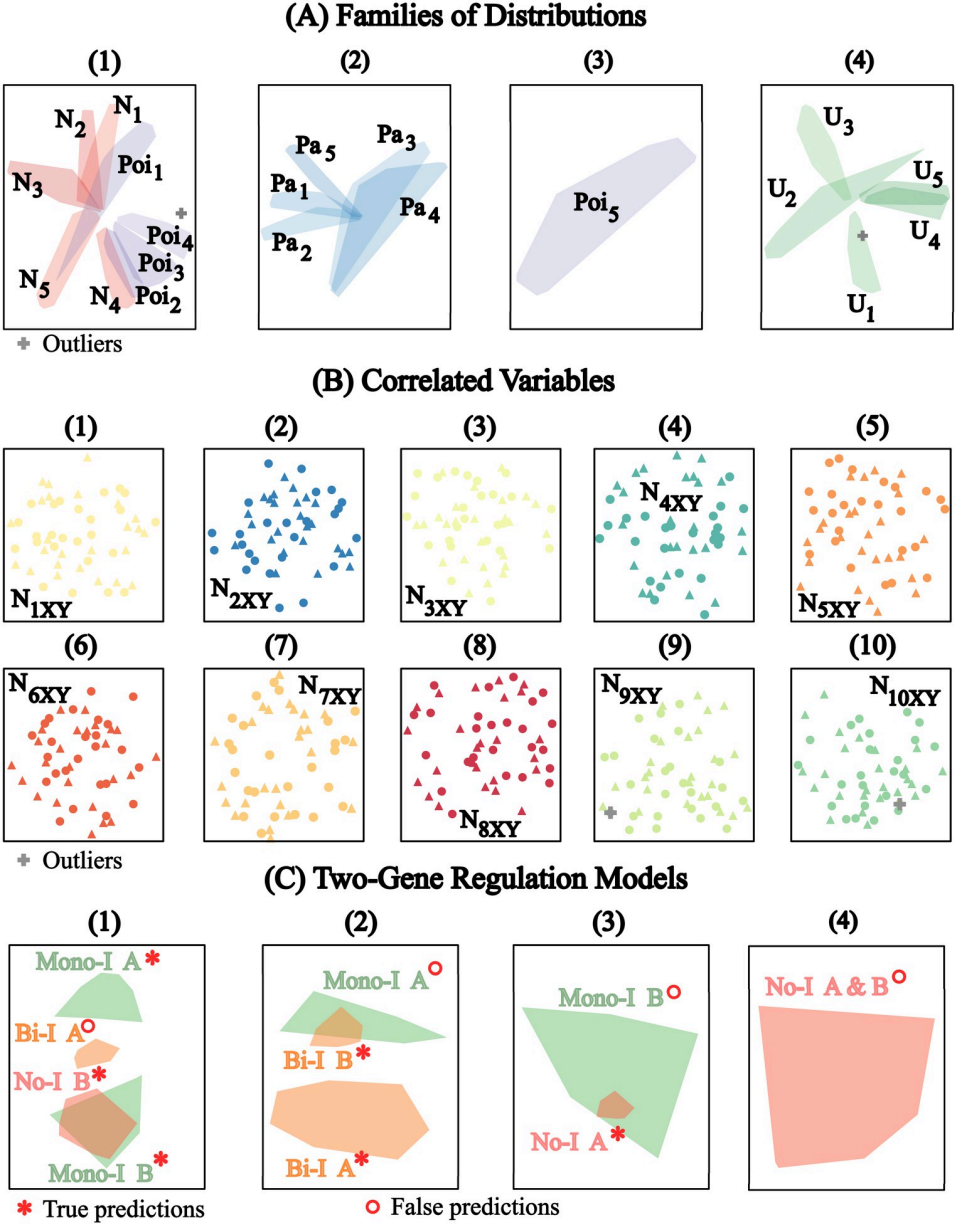
MIASA-prediction for *L*^3^-norm similarity distance. A: t-SNE projections of predicted clusters (in separate panels 1–4) versus convex hulls of data points belonging to the different distributions: Poisson (Poi_1_ to Poi_5_), Normal (N_1_ to N_5_), Pareto (Pa_1_ to Pa_5_), and Uniform (U_1_ to U_5_). B: t-SNE projections of predicted clusters (in separate panels 1–10) versus samples of bivariate normal distributions, first and second dimensions (N_1XY_ to N_10XY_). C: t-SNE projections of predicted clusters (in separate panels 1–4) versus true gene regulation patterns between gene A and gene B (convex hulls of data point representations): No-I A, No-I B, Mono-I A & B, and Bi-I A & B. True and False predictions are only evaluated for the pairs of genes belonging to the same models.

The results for the *L*^3^-norm similarity distance are shown in Fig 3 using t-SNE visualization. For the distribution datasets (Problem 1), we kept the *L*^3^-norm similarity distance for X and embedded Y to obtain the required Euclidean similarity distance corresponding to the *L*^3^-norms between the representations of the sample vectors (qEE-Transition: Dealing with a Non-Euclidean similarity). For the other problems, all similarity distances were transformed into Euclidean equivalents. The predicted family of distributions, shown in Fig 3A, were the same as for the Euclidean similarity distance (Fig 2A). The individual distributions are still well distinguished on the t-SNE projections although with rather elongated shapes. The predicted correlated variables are shown in Fig 3B and it also has an accuracy of almost 100% (only two outliers in clusters 9 and 10). The t-SNE maps, however, displayed a well-mixed projection of the sample vectors of the first and second dimensions of the correlated samples as opposed to the patterns seen in Fig 2B. For the two-gene interaction models, all true interaction models were also included in the result, however, with slightly more false predictions (Fig 3C) than in the case of the Euclidean similarity distance (Fig 2C). In terms of projection, the t-SNE also showed a clear separation between the genes of the Mono-I model but also the genes of the Bi-I model (Fig 3C panel 2).

### Performance evaluation: With qEE-Transition vs. without

**Fig 4:**
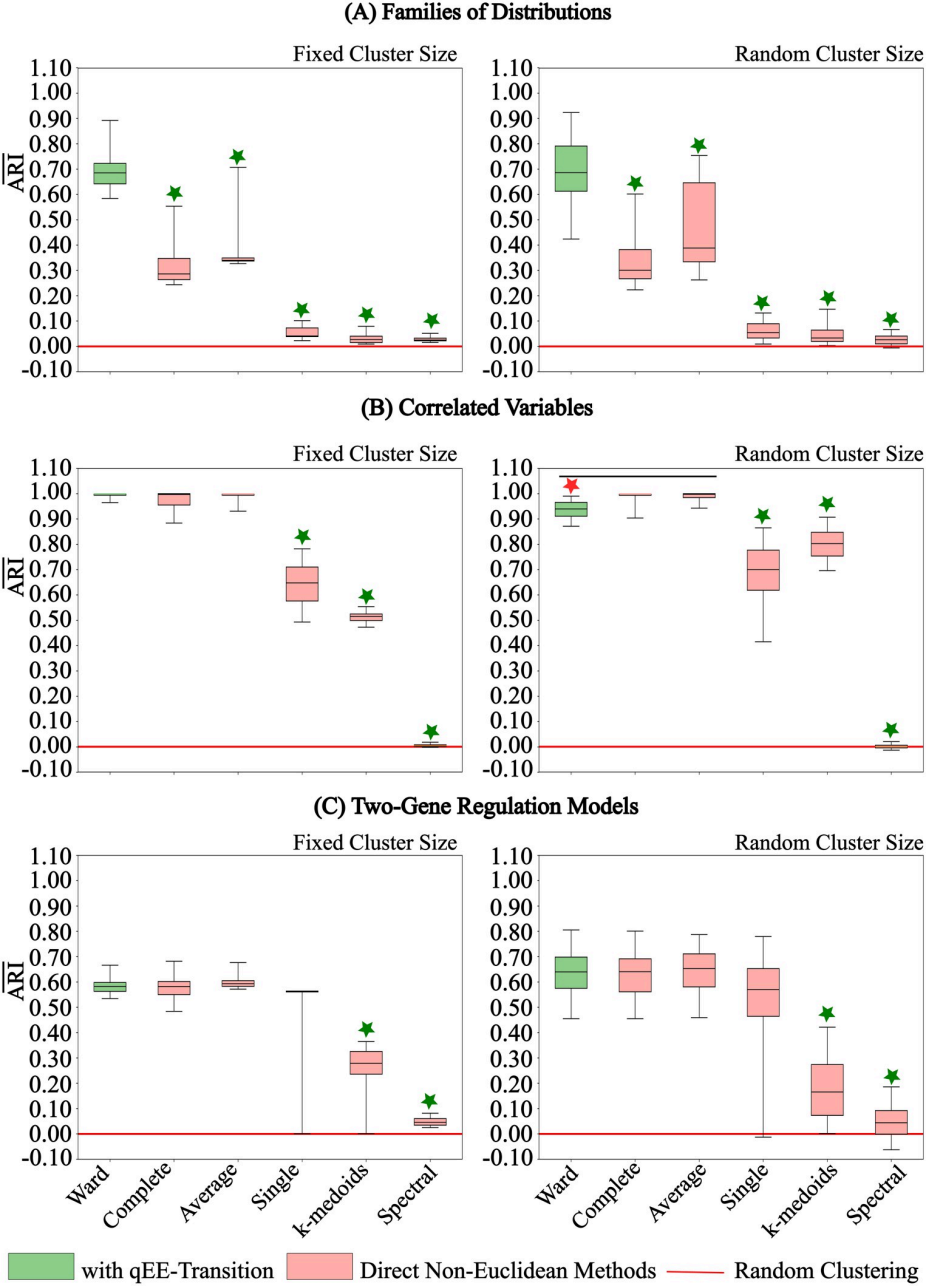
Accuracy of MIASA vs. Non-metric framework. Distribution of accuracy scores (Eq 15) for 2000 experiments for Euclidean similarity proximities and specific association distance (Potential applications). A: Prediction accuracy for families of distributions for fixed (25) and random (2 to 25) number of sample vectors per distribution. B: Prediction accuracy for bivariate normal correlated sample vectors with fixed (25) and random (2 to 25) pairs of sample vectors per distribution. C: Prediction accuracy for identification of regulation for two-gene regulatory network models with fixed (25) and random (2 to 25) pairs of gene representations sampled from each model. Whiskers indicate the 5^*th*^ − 95^*th*^ percentile of the score distribution and colored star indicates a significant Man Whitney U test corresponding to the significantly superior method (p-value <0.001 and *r* > 0.6).

We evaluated our contribution to the problem of data clustering by comparing the accuracy clustering results with the qEE-transition to the ones without. As a measure of accuracy, we adopted the adjusted rand index ($\bar{\text{ARI}}$) which provides a quality assessment score between the predicted cluster membership and the true cluster membership relative to a purely random clustering procedure (Performance Evaluation). For each investigated problem (Potential applications), we generated accuracy score distribution from 2000 experiments each time generating different datasets. Our evaluation results are presented in Fig 4 which shows that overall the clustering results with the qEE-Transition performed significantly better than the non-agglomerative methods (Man Whitney U test p-value <0.001 and *r* > 0.6) but had a varied performance against the agglomerative methods. For a fixed number of objects in each true distribution (Fixed cluster size), the qEE-Transition enabled a median accuracy of around 0.68 (IQR = 0.65, 0.75) whereas all the direct non-Euclidean-based methods had a median accuracy between 0 and 0.35 with a quite narrow IQR ranges except for the complete linkage method (Fig 4A left panel). This means that in 75% of the simulations, the MIASA framework performed 65% better than expected from a random clustering procedure whereas the direct non-Euclidean-based methods rather resulted in a random clustering of the objects. Our framework performed the best for the dataset of correlated random variables in which the accuracy score was narrowly distributed around 1 (Fig 4B, left panel). The direct non-Euclidean-based clustering methods, had varied performances, with the agglomerative method having accuracy scores distributed narrowly around 1, and the non-agglomerative methods having accuracy scores distributed between 0 and 0.5. The last dataset representing the two-gene regulatory network models had the lowest overall accuracy scores as compared to the other dataset types (Fig 4C, left panel), however, the result shows that the qEE-transition combined with Ward still performed at least 55% better than a random clustering procedure. The qEE-transition combined with Ward clustering and the non-Euclidean-based agglomerative methods had an accuracy between 0.55 and 0.7, and the non-agglomerative methods had an accuracy ranging between 0 and 0.35. Similar comparisons can be made in the case of a random cluster size (Fig 4 right panels), however, the ranges of the accuracy distributions are wider, indicating some sensitivity with the size of the true clusters.

### Experiments on machine learning methods

**Fig 5:**
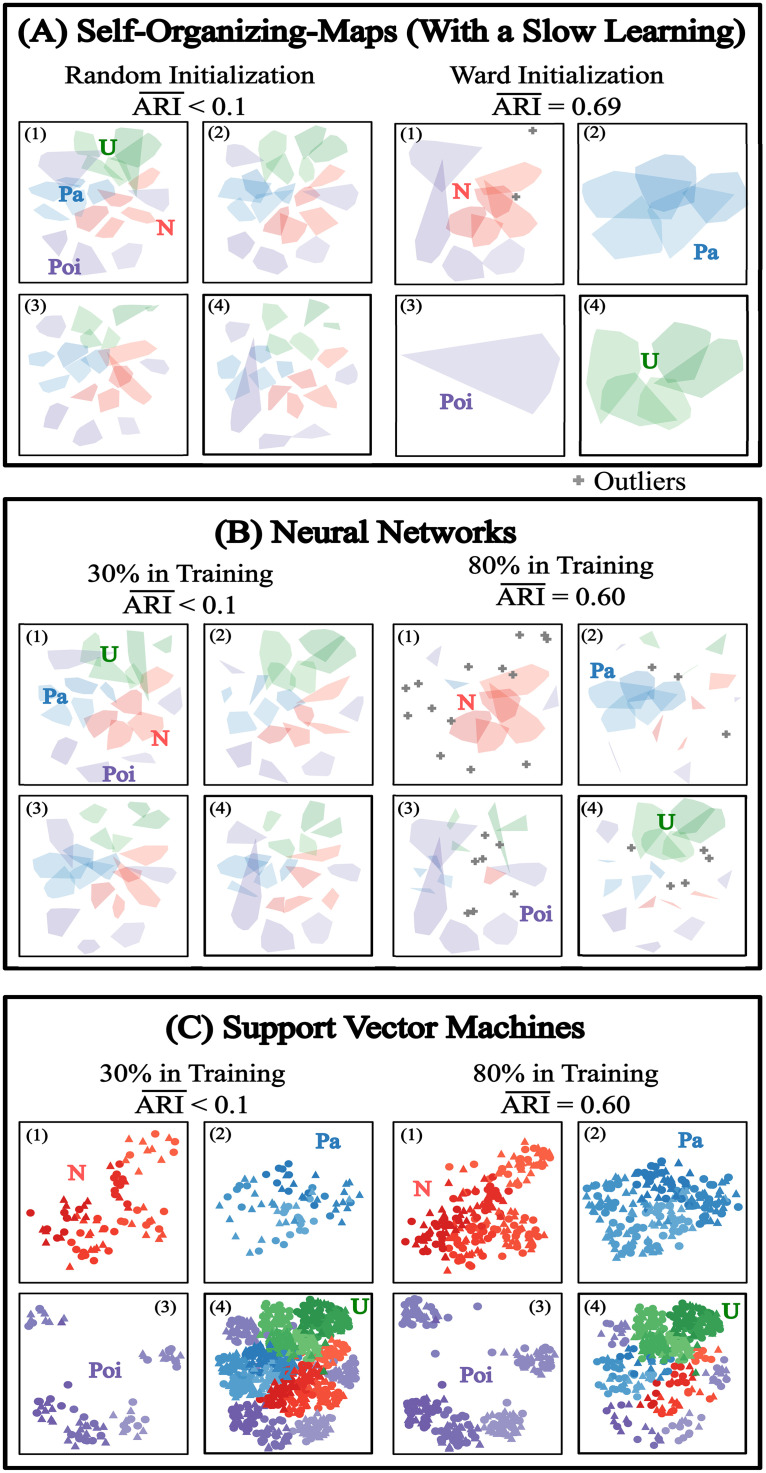
Experiments on machine learning methods. qEE transition combined with different Euclidean distance-based or vector-based machine learning methods applied to the distribution dataset (predicted clusters are shown in different panels as in Fig 2).

Due to the uncertainties in the optimal parametrization of the machine learning methods (see Clustering/Classification and lower dimensional visualization), we only conducted a few experiments with the distribution dataset. These experiments were performed after obtaining the joint Euclidean embedding of the separated datasets X and Y using the qEE-Transition. In all machine learning methods, we provided a fixed maximal number of iterations as three times the number of sample vectors, and unless specified here, for all other parameters we used the default setting provided by the Python package. For the Self-Organizing-Maps (SOM), we provided a slow learning rate parameter equal to $10^{-20}/\sqrt{c_{3}\zeta }$ and simulated two different initial conditions: one with a random initial condition and the other with the result of Ward clustering as an initial condition (Fig 5A). The results show that the SOM with the random initialization performed poorly with an $\bar{\text{ARI}}$ score bellow 1% and all the predicted clusters look similarly composed with some proportions of the true distributions (Fig 5A left panel group). With the Ward initialization, the SOM remained at the same prediction result (Fig 5A right panel group). This suggests that the SOM algorithm might struggle to overcome local optima. For the Neural Network (NN) and the Support Vector Machines (SVM), we simulated two proportion parameters for the training datasets: the first one uses 30% of the data as training and the second one uses 80% of the data as training (Figs 5B and 5C). Curiously, the prediction result of the NN with the 30% training closely resembles that of the SOM with random initialization (Fig 5B left panel group). With 80% training, the NN method achieves a good accuracy of 60%, however, the predicted clusters contain many outliers and small groups of other true cluster members (Fig 5B right panel group). Finally, the SVM method provided the best cluster structure despite having an accuracy measure similar to that of the SOM and the NN (Fig 5C). We can see that, in both cases, i.e., 30% training and 80% training, the SVM achieves similar prediction structures. The 30% training has a poor accuracy only because there is a denser cloud of points in the fourth cluster (Fig 5C left panel), however, with 80% training, the clouds of points become denser in the well-predicted clusters (1, 2, 3) and thinner in the fourth cluster which leads to a much higher accuracy measure (Fig 5C right panel). Qualitatively, thus, with our parameter settings, the SVM-predicted cluster is much better than the NN- and SOM-predicted clusters.

## Discussion

In this paper, we presented MIASA, a framework that enables the joint analysis of distinct variables describing the same phenomenon or process and which can be associated with multiple different information defining their interaction. Here, a distinction is made between the terms similarity vs. association (despite that they could be used interchangeably) because it provides a simple way of presenting the notion of multiple-interaction measures. For simplification we formulated the problem using two variables, however, it can be extended to any number of variables by properly re-distributing the datasets between the conceptual datasets X and Y (as can already be seen with the distribution data example in Potential applications). Our framework starts by assembling user-defined similarity vs. association distances, then, it transforms the assembled distance matrix into an equivalent Euclidean distance (qEE-Transition) and completes the clustering process by applying an Euclidean-distance-based clustering/classification method. The construction of the required Euclidean distance is based on a qualitative Euclidean embedding (qEE) method which requires that at least one similarity distance is Euclidean [28]. However, any arbitrary similarity distance can also be used because the qEE method can also construct an Euclidean distance equivalent to it. As the Euclidean distance-based clustering method, we selected the agglomerative hierarchical clustering with Ward’s linkage method for the accuracy comparison because it performed the best, in combination with the qEE-Transition, during our numerous test simulations. However, in the implementation of our framework, we included the possibility of collecting the qEE-transformed dataset, in standard machine-readable formats, enabling all users to apply any other clustering or machine learning methods of their choice (several clustering and machine learning methods are also integrated into the framework pipeline).

As an alternative to conventional optimization-based non-metric multidimensional methods which do not necessarily preserve the distance information and require a prior input on the number of dimensions, our qEE-Transition strictly preserves the distance information. It is also not concerned with dimension reduction but rather finds an appropriate Euclidean space that provides the distance information. The dimension of the object in their respective original feature space is not important in our framework because the dimension of the Euclidean embedding space tends to always be equal to the number of points that need to be embedded. This means that, in terms of reduced dimension, our method provides an advantage when the dimensions of the original feature spaces of the datasets are much larger than the number of objects. We want to stress, however, that the qEE-Transition encodes the assembled distance matrix in the Euclidean distance between the embedded coordinates and not in any other distance measure. Therefore, using a non-Euclidean distance derived from the qEE coordinates of the dataset objects as a distance basis for the clustering process might not provide the pattern corresponding to the originally assembled similarity and association information. In a sense, our framework unifies all distance-based clustering/classification problems into the Euclidean distance-based clustering/classification problem. Therefore, the clustering/classification performance will improve alongside new developments in Euclidean distance-based clustering and machine learning methods.

We used three different datasets to illustrate the performance of our approach: families of distributions, correlated random variables, and two-gene regulatory network models. For predicting the families of distributions, the numerical histograms of the samples defined similarity distance, and the Kolmogorov-Smirnov p-value defined the association distance. This assumption provided a good prediction pattern for families of distribution albeit a mixing of the Poisson and the Normal families. Additionally, the UMAP projection showed that individual distributions are still quite well-separated suggesting the information on the individual distributions was well captured by the association distance. Therefore, it should be possible to separate the Normal and the Poisson families by further analyzing the patterns in the predicted clusters. The dataset of correlated samples was the easiest to reconstruct. This indicates that the choice of combination of the similarity and association distances used was well in line with the definition of correlation. The third dataset, composed of three two-gene interaction models was moderately reconstructed. The Euclidean similarity distance provided better results than the *L*^3^-norm similarity distance. The two genes of the bidirectional interaction model (Bi-I) and the mono-directional interaction model (Mono-I) were correctly predicted as belonging to the same cluster whereas the two genes of the no-interaction models displayed both true and false predictions. Additionally, the UMAP projection suggests that the genes of the Bi-I model are well mixed, the genes of the Mono-I models are well separated, whereas the genes of the No-I model are more disorganized. This pattern may be explained by the mixing of regulation and correlation in the different models. The No-I model purely contained correlation seen as an overlapping upward trend in the mean mRNA counts, the Mono-I model included a mixture of regulation and correlation also seen as an upward trend on the mean mRNA counts but lower for gene A since gene A was down-regulated by gene B, and the Bi-I model purely contained regulation albeit it is also seen as an overlapping upward trend in the mean mRNA counts [37]. The trends in the mean mRNA counts mean that the No-I model and the Bi-I model are the same and this explains the similar mixing of the genes between the No-I and the Bi-I model, however, since there is no regulation in the No-I model, the genes where also identifies separately in other clusters. As for the Mono-I model, the clear separation between the genes in the UMAP projection is also explained by the moderate regulation pattern. Our gene-regulation clustering pattern indicates that the Granger causality association distance measure effectively identifies regulation but struggles to differentiate between correlation and regulation. However, the regulating genes seem to be consistently predicted within the same cluster, thus, the consistency of the combination of the genes might provide a cut between regulation and correlation.

Our accuracy analysis (Performance evaluation: with qEE-Transition vs. without) indicates the robustness of the above assessment and the potential advantage that the qEE-Transition might provide over the direct non-Euclidean clustering methods. From this performance assessment, we can conclude that the qEE-Transition combined with the Ward clustering method had a significant advantage as compared with non-agglomerative methods. Our method had similar performance when compared with the agglomerative methods except for the distribution data where it performed significantly better. Our results are sensitive to the scaling between similarity vs. association distance. This is seen in the change in the cluster structure of results based on different scaling of the distances (S1 Fig). Care should thus be taken when choosing any scaling of similarity vs. association distance because the clustering algorithm might become biased toward either of them. At this point, we are not sure about the appropriate scaling trends. However, we recommend that the users test out several scaling options according to which distance information they need to prioritize over the other.

As a novel contribution to the data classification process in machine learning, our framework offers to make Euclidean-distance- or vector-based machine learning available for classifying disjoint datasets (Experiments on Machine Learning Methods). Similarly to the clustering methods, this is possible due to the mediation of our information-preserving qEE-Transition. Our few experiments on the distribution data suggested that the Support Vector Machines method provided the best classification results in terms of accuracy measure and cluster structure. This result is, of course, dependent on our parameterization. We did not include machine learning methods as part of the accuracy analysis because of the lack of proper optimization of parameter choice. To execute other parameter-optimized methods, all users can easily collect the qEE-transformed dataset by running our pipeline on their dataset. In addition, our machine learning results were obtained only because of the qEE-Transition, however, purely technically speaking, the rows of the assembled distance matrix could also be used as a vector representation of the sample vectors. This would represent the assumption that the pairwise interaction between all the objects involved is a function of all the other pairwise interactions within the dataset. Nevertheless, we did not include this possibility as an option for comparing our method because we find this assumption difficult to justify, and because our tests showed that this would provide a poor structure of predicted clusters or a non-increasing learning performance on the different training data (see S2 Fig). This suggests that the assumption of using pairwise distances as Euclidean vector representation might not be an appropriate choice and reinforce the contribution of the qEE-Transition.

In terms of visualization, the high dimensionality of the qEE-transformed dataset did not allow us to perform the standard two-dimensional PCA projections (orthogonal projection). Therefore, it is currently not possible to thoroughly visualize the pairwise interaction between the elements of the different datasets. Here, we used non-linear dimension reduction methods such as UMAP and t-SNE, however, these methods also do not provide full interpretability of the pairwise interpoint distance between the projections of the datapoints. Therefore, more work is needed to properly reduce the dimensionality of the qEE and allow a detailed analysis of pairwise interactions. Additionally, our experiments here are certainly not exhaustive because of the large number of clustering methods and distance definitions that have been already developed. We focused mainly here on presenting the MIASA framework and providing a snapshot of potential applications in data analysis. One advantage of our framework is that it bridges the gap between Euclidean-distance-based and non-Euclidean-distance-based clustering and machine learning methods. In other words, it unifies all distance-based clustering/classification problems into the Euclidean-distance-based problem. Therefore, knowledge-driven choice of similarity and association distance is the most crucial step in recovering structures within datasets. The generic approach for identifying appropriate clustering methods for the dataset consists of testing all different types of distances and cluster validations scores (e.g., [12, 18, 51]). However, each distance measure carries a specific type of information that might or might not be appropriate for the dataset or the phenomenon that they describe. By improving the clustering results and enabling machine learning methods for disjoint datasets, our framework helps to assess how relevant assumptions on the interaction distance are, to the investigated phenomenon.

Our framework points to the importance of distance distribution models. This type of model has already been investigated for general *L*^*p*^− metrics to optimize the performance of nearest-neighbor methods applied to investigate similarity or associations between representations of the same variable [52]. In the future, our framework can support the development and validation of interaction-distance distribution models to capture complex interactions between distinct variables. As shown in [52], regardless of the underlying data distribution, the pairwise Euclidean distance, between pairs of samples, asymptotically tends to a normal distribution as the number of dimensions of the feature space increases. Though some adjustment might be necessary for jointly distributed random variables (as we might think of interacting distinct variables), our qEE-Transition derives a new high dimensional feature space that only depends on the number of objects involved in the analysis. Therefore, for a large enough number of objects in each disjoint dataset, the qEE-Transition derives a distance distribution close enough to a normal distribution regardless of the original interaction-distance distribution to which the assembled distance matrix belongs. Exploring the qEE-transform dataset from the perspective of Euclidean distance-distribution models might provide insights into the original interaction-distance distribution and the complex interactions between the variables involved.

## Conclusion

In summary, this paper presents the MIASA framework for a holistic analysis of the multiple interaction patterns between distinct variables represented in disjoint datasets through the mediation of an information-preserving transformation (qEE-Tansition). These interaction patterns are conceptualized as similarity when measured between the same variable and association when measured between different variables. We implemented our framework in Python enabling any users to apply most of the clustering algorithms and machine learning methods used in this paper and to collect the qEE-transformed dataset in standard machine-readable formats to apply their own clustering or machine learning methods. As a showcase, we applied our framework to three potential problems to show that the qEE-Transition method works well when combined with the Ward clustering and the Support Vector Machines methods, however, a better parameterization of the machine learning methods might improve the results. Here, we are not claiming to solve all the complex aspects of multi-variable data analysis because defining appropriate similarity vs. association measures is complex enough. However, we believe that our framework is simple and intuitive enough to enable researchers in any field involving data analysis to identify important information from multiple datasets. At this point of our research, the qEE-Transition method cannot be combined with orthogonal projection, however, in the future, we will investigate the possibility of refining the method to provide a PCA-like analysis of the pairwise interactions pictured by the assembled distance matrix. Additionally, our framework points to the importance of more developments and validation of distance-distribution models to capture complex interactions between disjoint variables especially when the interaction-distance information is available.

## Supporting information

S1 Fig MIASA-prediction for scaled distances. A: Families of distributions with max-scaled histograms representations, UMAP projections of predicted (in separate panels 1-4) versus convex hulls of the data points belonging to the different distributions: Poisson (Poi_1_ to Poi_5_), Normal (N_1_ to N_5_), Pareto (Pa_1_ to Pa_5_), and Uniform (U_1_ to U_5_). B: Correlated variables with (1/2)-scaled association distance, UMAP projections of predicted (in separate panels 1-10) versus samples of bivariate normal distributions, first dimensions (N_1X_ to N_10X_) and second dimensions (N_1Y_ to N_10Y_). C: Two-Gene Regulation Network with (1/2)-scaled similarity in gene A, UMAP projections of predicted (in separate panels 1-4) versus true gene regulation patterns between gene A and gene B (convex hulls of data point representations): No-I A, No-I B, Mono-I A & B, and Bi-I A & B. True and False predictions are only evaluated for the pairs of genes belonging to the same models.
(TIF)

S2 Fig Experiments on machine learning methods using distances as features. Test results when the rows of the assembled distance matrix are used as Euclidean configuration of the sample vectors for the distribution dataset (without the qEE-Transition step).
(TIF)
