# Supplementary figures and images for: Multi-Input data ASsembly for joint Analysis (MIASA): A framework for the joint analysis of disjoint sets of variables
Source: PLoS One. 2024 May 10;19(5):e0302425. doi: 10.1371/journal.pone.0302425 (PMC11086896; doi:10.1371/journal.pone.0302425)

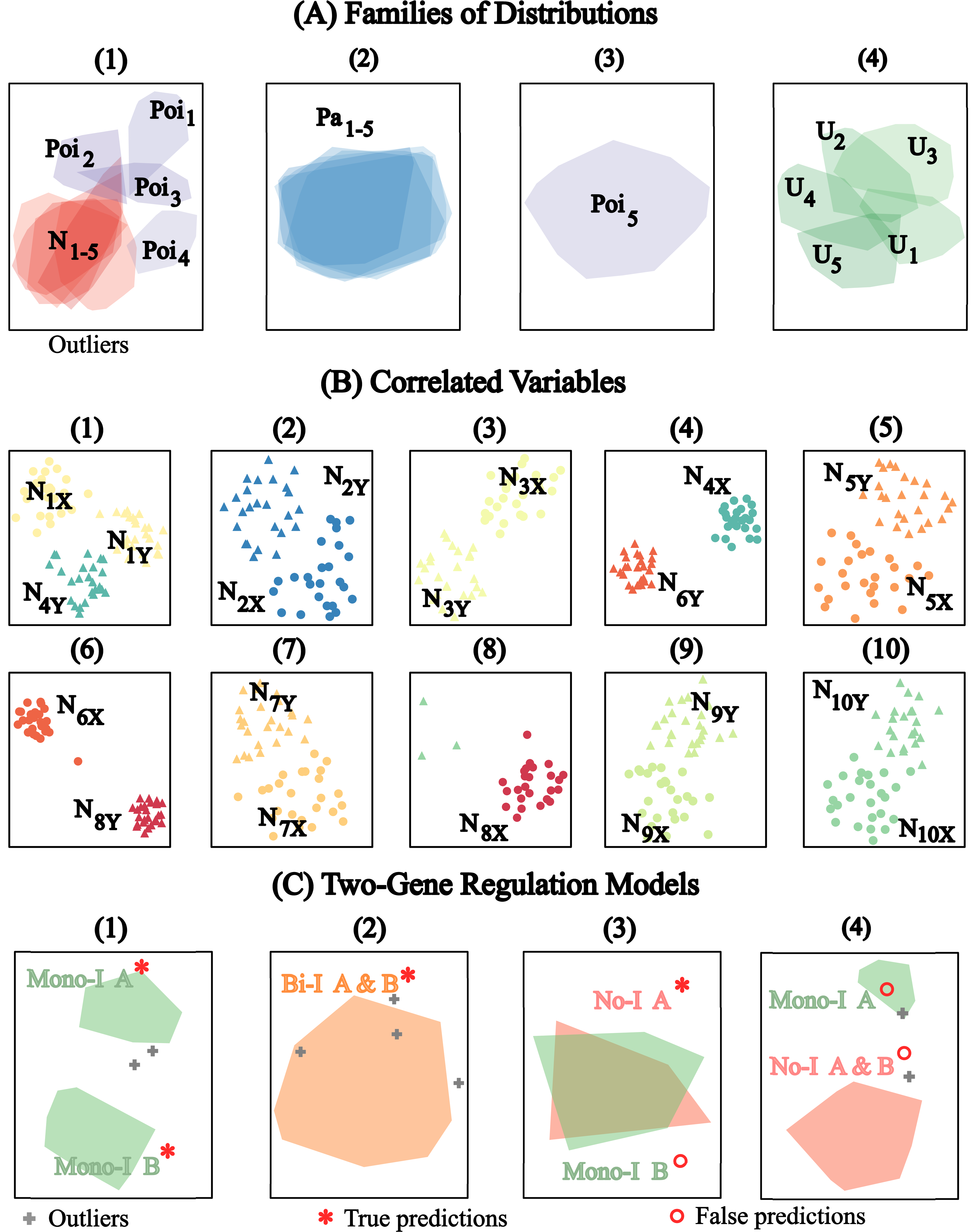

Supplement: S1 Fig — A: Families of distributions with max-scaled histograms representations, UMAP projections of predicted (in separate panels 1-4) versus convex hulls of the data points belonging to the different distributions: Poisson (Poi1 to Poi5), Normal (N1 to N5), Pareto (Pa1 to Pa5), and Uniform (U1 to U5). B: Correlated variables with (1/2)-scaled association distance, UMAP projections of predicted (in separate panels 1-10) versus samples of bivariate normal distributions, first dimensions (N1X to N10X) and second dimensions (N1Y to N10Y). C: Two-Gene Regulation Network with (1/2)-scaled similarity in gene A, UMAP projections of predicted (in separate panels 1-4) versus true gene regulation patterns between gene A and gene B (convex hulls of data point representations): No-I A, No-I B, Mono-I A & B, and Bi-I A & B. True and False predictions are only evaluated for the pairs of genes belonging to the same models. (TIF) [file pone.0302425.s001.tif]

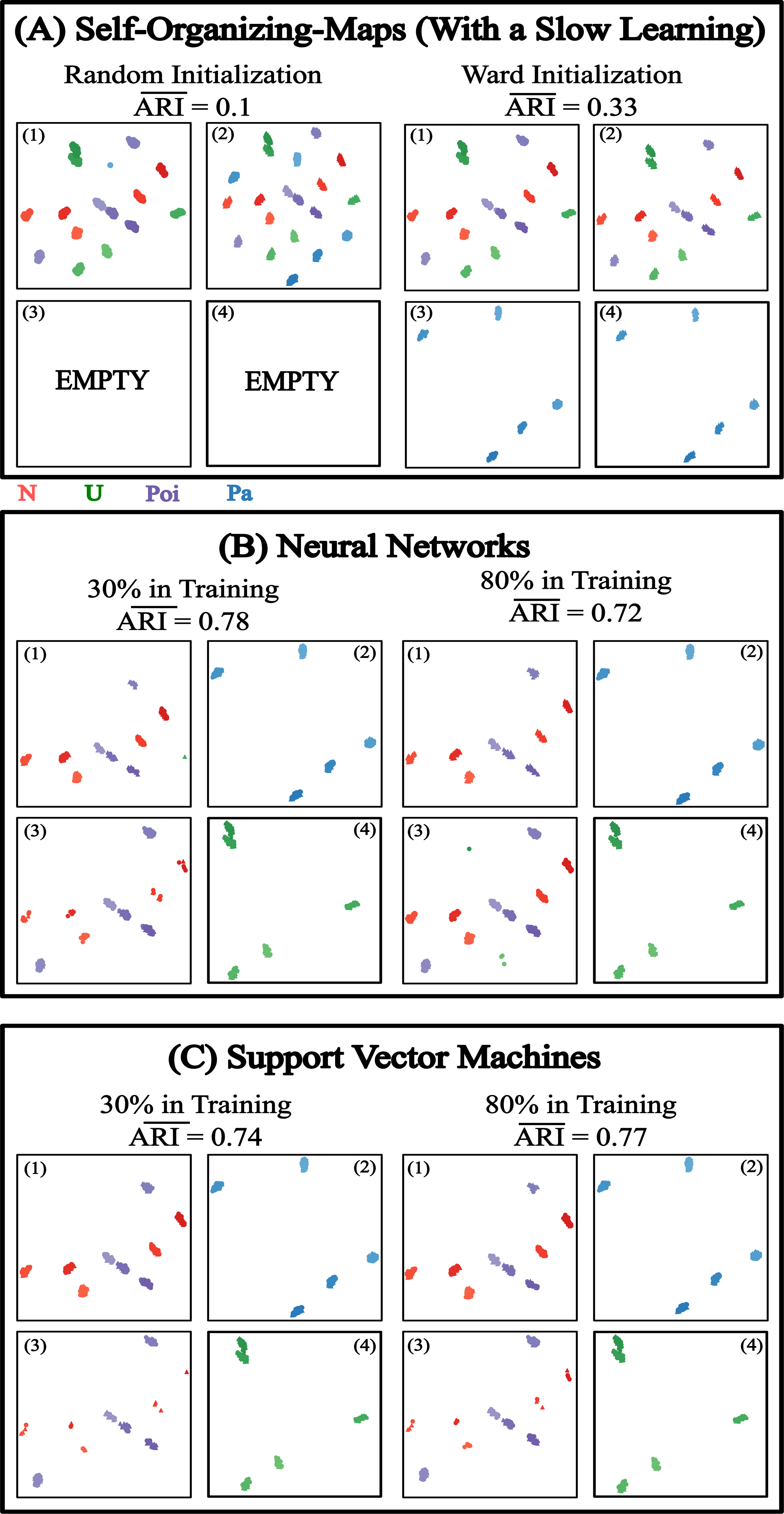

Supplement: S2 Fig — Test results when the rows of the assembled distance matrix are used as Euclidean configuration of the sample vectors for the distribution dataset (without the qEE-Transition step). (TIF) [file pone.0302425.s002.tif]
